# Reliable replicative lifespan determination of yeast with a single-channel microfluidic chip

**DOI:** 10.1242/bio.060596

**Published:** 2024-11-26

**Authors:** Valentina Salzman, Moises R. Bustamante Torres, Francisco G. Correa Tedesco, Nahuel Tarkowski, María J. Godás Willems, Joaquín N. Bravo, Magalí Mercuri, Dante G. Mercado, Guido Berlin, Martín G. Bellino, Pablo S. Aguilar, Laura C. Estrada

**Affiliations:** ^1^ Instituto de Fisiología, Biología Molecular y Neurociencias (IFIBYNE), CONICET. Universidad de Buenos Aires, Buenos Aires C1428EGA, Argentina; ^2^ Instituto de Investigaciones Biotecnológicas, Universidad Nacional de San Martín, San Martín 1650, Argentina; ^3^ Universidad de Buenos Aires, Facultad de Ciencias Exactas y Naturales, Departamento de Física. Buenos Aires C1428EGA, Argentina; ^4^ Departamento de Micro y Nanotecnología, Comisión Nacional de Energía Atómica (CNEA), Av. Gral. Paz 1499, San Martín, Buenos Aires B1650LWP, Argentina; ^5^ Instituto de Nanociencia y Nanotecnología (INN, CNEA-CONICET), Av. Gral. Paz 1499, San Martín, Buenos Aires B1650LWP, Argentina; ^6^ CONICET - Universidad de Buenos Aires, Instituto de Física de Buenos Aires (IFIBA). Buenos Aires C1428EGA, Argentina

**Keywords:** Microfluidics, Replicative lifespan, Clogging mitigation

## Abstract

S*accharomyces cerevisiae* is a powerful model for aging research due to its short lifespan and genetic malleability. Microfluidic devices offer an attractive approach enabling rapid monitoring of hundreds of cells during their entire replicative lifespan (RLS). Yet, key operational issues such as contaminations, cell loss, and cell-aggregates-dependent flow obstruction can hinder RLS experiments. We report the development of a microfluidic device configuration that effectively prevents flow blockage. We conducted comprehensive performance characterization, evaluating trapping efficiency, cell retention, budding orientation, and cell aggregate formation. The optimized device successfully supported long-term culturing and reliable RLS measurements of budding yeast strains. For accurate lifespan determination, a detailed workflow is provided that includes device fabrication, live microscopy setup, and characterization of cell age distribution. This work describes an accessible and reliable microfluidic device for yeast RLS studies, promoting further exploration in aging research.

## Teaser

**Summary:** Quantitative characterization of microfluidic devices for reliable and informative replicative lifespan analysis of budding yeast

## INTRODUCTION

*Saccharomyces cerevisiae*, the budding yeast, stands as an established model organism in aging research due to its short lifespan and the remarkable amenability of its genome to manipulation (He et al., 2018). As the first eukaryote with a fully sequenced genome, it has proven invaluable in identifying evolutionarily conserved aging-regulatory pathways (Longo et al., 2012; Taormina et al., 2019). Aging is quantified by replicative lifespan (RLS), which determines the finite number of daughter cells a single mother cell can produce through the process of mitosis. Due to cell clustering, accurate RLS determination requires the continuous removal of daughters for proper mother-cell tracking. The traditional assay (Mortimer and Johnston, 1959) involves growing isolated cells on agar plates. Here, a micromanipulator (a specialized microscope with a dissection needle and movable stage) facilitates the removal of mature daughter cells. This method is limited in throughput and is time-consuming, restricting studies to approximately 300 cells per experiment. For logistical reasons, the standard practice involves storing cells overnight at 4°C, extending the experiment to 4 weeks. Moreover, temperature fluctuations may introduce unintended effects on the experimental outcomes (Steffen et al., 2009). Most critically, the traditional assay significantly hinders the tracking of molecular markers within mother cells throughout their lifespan.

Several microfluidic devices (or chips) have been specifically designed to automate the microdissection process in yeast lifespan studies (Chen et al., 2017). Cultivation of single cells is achieved under precisely controlled environmental conditions, while daughter cells are removed by the fluid flow (Chen et al., 2017; Jo et al., 2015; Liu et al., 2015). Time-lapse microscopy within the chip enables real-time monitoring of cell budding over 3-4 days of continuous culture in standardized growth conditions. Moreover, by coupling molecular genetics with fluorescence live-cell imaging of chips, it is now possible to probe single yeast cell responses over a lifetime, to characterize population heterogeneity, and to study the underlying mechanisms of aging (Crane et al., 2019; Kukhtevich et al., 2022; Paxman et al., 2022).

Fabrication of microfluidic devices typically relies on soft lithography, a rapid prototyping technique well suited to iterative design (Xia and Whitesides, 1998). The most prevalent material choice is polydimethylsiloxane (PDMS) bonded to a glass substrate, owing to its biocompatibility, transparency, and ease of fabrication. The standard workflow for using these chips involves a specific sequence of essential steps: (1) design and fabrication, (2) preparation of cells and culture medium, (3) cell loading, (4) cultivation with integrated live-cell imaging, and finally, (5) data analysis. In particular, effective microfluidic device design requires careful consideration of several interconnected parameters: efficient cell capture, sustained nutrient delivery for long-term cultivation, and most importantly prevention of chip clogging, a critical factor that can negatively impact all aforementioned aspects. Unwanted cell proliferation outside designated areas is a persistent challenge in RLS microfluidic experiments (Täuber et al., 2021). Cell clumps are frequently observed around the inlet, outlet, and even within the internal device structures. These aggregates can significantly disrupt the intended flow profile, thereby compromising nutrient supply and potentially leading to complete flow cessation within the chip. While the impact of microfluidic device design on different parameters used to characterize experimental success, such as cell trapping or retention rates, is well-documented (Jo et al., 2015; Täuber et al., 2021), rigorous analysis is necessary to establish a precise correlation between specific geometric features and chip clogging by cell aggregates.

In this study, we report the development of a microfluidic device specifically designed to overcome the challenge of undesired cell growth locations during RLS determination. Two trap dimensions were employed, both exhibited high efficiency in cell retention and downstream bud orientation, which facilitates daughter-cell dissection. The proposed chip features a trap-grid configuration to ensure successful long-term culturing without clogging. Long-term yeast culturing and time-lapse imaging over 90 h revealed accurate and reproducible RLS measurements. We provide a detailed workflow that enables reliable fabrication of microfluidic devices at low cost, setup of long-term RLS experiments, and obtention of accurate yeast lifespan determination.

## RESULTS

### Design and fabrication of microfluidic devices

**Fig. 1.**
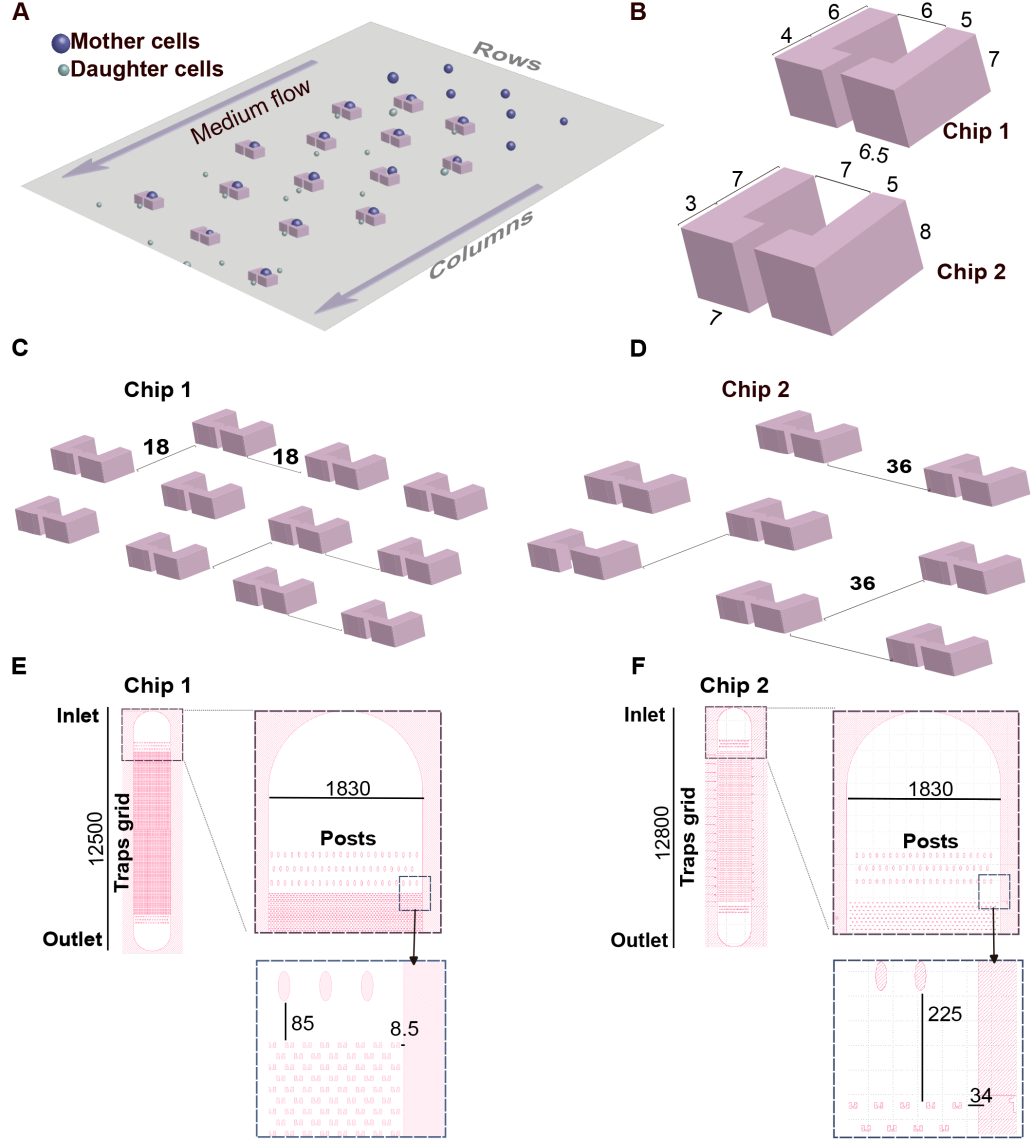
**Optimizing microfluidic devices designed for long-term maintenance of yeast cells.** (A) Schematic diagram of the overall structure showing the chip's working principle. Trap rows and columns are indicated. L-shaped microtraps were designed with dimensions indicated in B and intertrap distances shown in C and D. (E,F) Grid in chip 1 has 20,520 traps (380 rows×54 columns) whereas in chip 2 has 5882 traps (173 rows×34 columns). Oval-shaped posts positioned before and after the traps grid are designed to provide structural support to the wide microfluidic channel, preventing collapse during chip bonding. Dimensions are expressed in µm. All design work was done in Klayout software (https://klayout.de/).

To achieve reliable long-term culturing and microscopic observation of *S. cerevisiae* cells, we designed and fabricated microfluidic devices in two different geometries. The chips feature integrated traps that enable the capture and retention of individual mother cells while facilitating their outward budding (Crane et al., 2014; Jo et al., 2015; Wang et al., 2022). A continuous fluid flow confines mother cells within the traps, effectively removing daughter cells downstream (Fig. 1A).

Each trap consists of two opposing L-shaped pillars, with a central small outlet opening. This design exploits the characteristic axial budding pattern of *S. cerevisiae* haploid cells, where each daughter cell emerges adjacent to the previous one (Chant and Pringle, 1991). This pattern enables straightforward enumeration of daughter cells as they exit the trap through the small outlet. However, for mother cells that bud upstream, multiple daughters may accumulate within the larger opening, making cell counting unreliable. We decided to explore variations in trap size to achieve the following goals: (1) the trapped cells are stably kept throughout the aging experiment; (2) the trap does not pose a spatial constraint to cell size increase during aging and thus properly alleviates structural compression (Gao et al., 2020); and (3) the trap provides free space for cell rotation so that cells re-orient their buds towards the downstream side. To meet the aforementioned criteria, we devised two chip geometries (Fig. 1B).

Increasing trap dimensions might offer two potential benefits. First, the reported capacity of elongated traps to facilitate cell reorientation in diploid cells (Wang et al., 2022), suggests that larger trap dimensions could enhance the ratio of mother cells producing daughters that exit through the smaller outlet. Second, larger traps may mitigate the likelihood of cell expulsion caused by aging-related cell volume increase, thereby reducing compression stress. While both traps can accommodate young haploid cells (4-5 µm in diameter), chip 1 features significantly smaller trap dimensions than chip 2.

Another critical design feature of the chip is the spatial arrangement of the traps. Both chips feature a grid-like trap layout, with media flowing through the columns formed by the traps (Fig. 1A). Traps within each column are strategically positioned to align precisely with the center of the gaps between traps in adjacent columns. This configuration optimizes trapping efficiency and provides space for daughter-cell removal (Wang et al., 2022). Critically, the efficiency of cell removal depends on the distance between traps. Yeast microfluidics devices can become clogged due to the formation of microcolonies through the progressive accumulation of mother-daughter cell aggregates (Crane et al., 2014; Jo et al., 2015; Liu et al., 2015; Richter et al., 2022). Moreover, large, dying cells can accelerate clumping and microcolonies formation. This poses a significant concern, as it not only diminishes the number of analyzable regions within the chip but can also disrupt the experimental flow, potentially halting the experiment altogether. Larger inter-trap distances mitigate chip clogging (Jo et al., 2015), albeit at the expense of reducing the density of traps per unit area. Consequently, to minimize cell accumulation outside traps, we evaluated inter-trap distances of 18 μm and 36 μm for chip 1 and chip 2, respectively (Fig. 1C,D).

Both chips hold a single microchannel that includes an inlet space for cells and medium infusion, the traps grid, and an outlet space for waste disposal (Fig. 1E,F). Following the inlet and before the outlet, there are oval-shaped posts designed for support and to prevent the collapse of the wide microchannel's ceiling. Positioned between the supporting posts the grids comprise 20,520 and 5882 traps in chip 1 and chip 2, respectively. The spatial separation between the trapping array and the posts is approximately fourfold greater in chip 2 than in chip 1 (Fig. 1E,F). This increment is similarly reflected in the distance from the edge trap columns to the lateral limits of the microchannel. We reasoned that increasing the free space in these regions would facilitate the removal of daughter cells and reduce clogging.

Glass chrome photomasks containing each design were used to fabricate master molds by photolithography using a SU-8 2005 photoresist (see Materials and Methods). The molds’ thicknesses (as a proxy of the trap's height) were determined using a mechanical profilometer (Fig. S1). The resulting PDMS chips replicate the master molds and feature the microchannel upon bonding to a glass coverslip (Fig. S2). A detailed fabrication protocol for PDMS chips is described in the Materials and Methods.

### Performance characterization of cell traps

To evaluate the performance of the microfluidic devices, we employed a microfluidic system that enabled simultaneous operation of two RLS assays (chip 1 and chip 2). Chips were first loaded with mid-log phase yeast cell cultures and then kept under continuous growth media flow using a syringe pump. Subsequently, the chips were mounted onto the microscope stage and time-lapse images were captured at various positions across the grid (Fig. S3). RLS assays were considered valid if they ran continuously for a minimum of 70 h. We chose this threshold based on the minimum experimental time required to accurately monitor the lifespan breadth of a population of *S. cerevisiae* haploid wild-type (WT) cells (Jo et al., 2015).

**Fig. 2.**
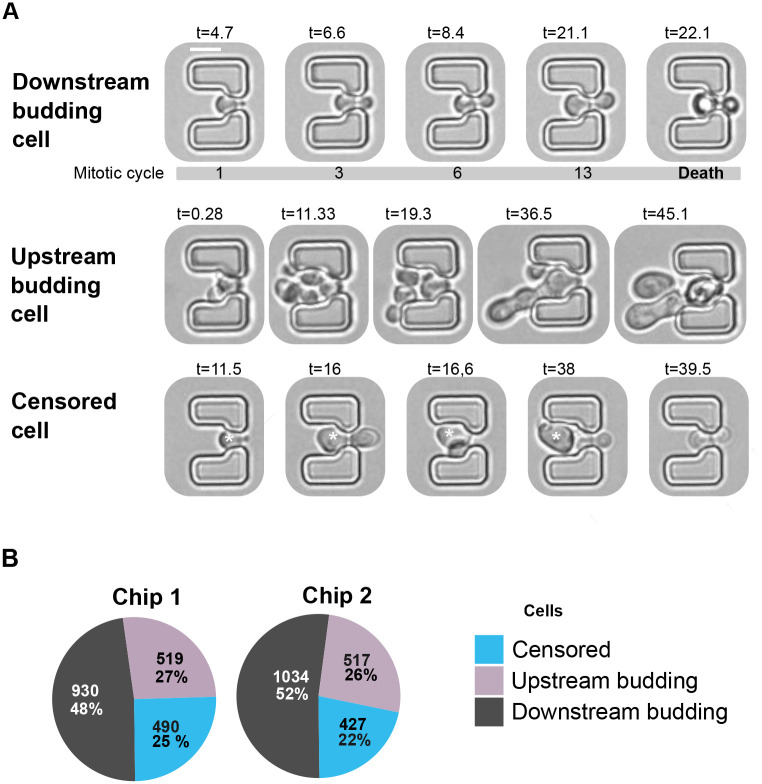
**Performance characterization of traps for monitoring yeast RLS.** (A) Bright-field images exemplifying downstream budding, upstream budding, and censored cells. Time (t) since the RLS microfluidic experiment started is indicated in h. Scale bar: 5 µm. The censored cell is marked by an asterisk. (B) Proportions and percentages of downstream budding, upstream budding, and censored cells were identified in a total of 1939 and 1978 trapped cells in chips 1 and 2, respectively.

Based on their behavior in the traps, we classified the monitored mother cells into three groups: (1) downstream budding cells (cells budding towards the smaller outlet opening and remaining trapped throughout the experiment), (2) censored cells (downstream budding cells that breached the trap before 70 h) and (3) upstream budding cells (cells budding counter to the flow direction) (Fig. 2A). The first group of cells (1) is the population relevant for RLS determinations. To ensure our analysis focused on cells with full lifespan potential, we only included those trapped within the first 20 h of the experiment. This criterion excluded cells that might have originated from aged mother cells, potentially inheriting a limited lifespan (Jo et al., 2015; Kennedy et al., 1994).

We ran four independent RLS experiments with chips 1 and 2 operating in parallel. After monitoring approximately 2000 cells per chip type, our results show that the majority of traps (approximately 70%, including censored cells) held downstream budding cells, highlighting the effectiveness of the microfluidic designs (Fig. 2B). This observation suggests that within both chips, there exists substantial space for cells to get placed in alignment with the flow direction. Furthermore, the percentage of cells that escaped from traps also remained comparable between both proposed geometries (approximately 20%). These results indicate that both microfluidic chip designs are suitable for RLS experiments. However, chip 1 presents a distinct advantage by offering a higher density of traps per unit area. This increased density translates to the potential for analyzing a larger number of cells within a single experiment.

### Enhancing daughter cell removal by optimizing trap spacing

**Fig. 3.**
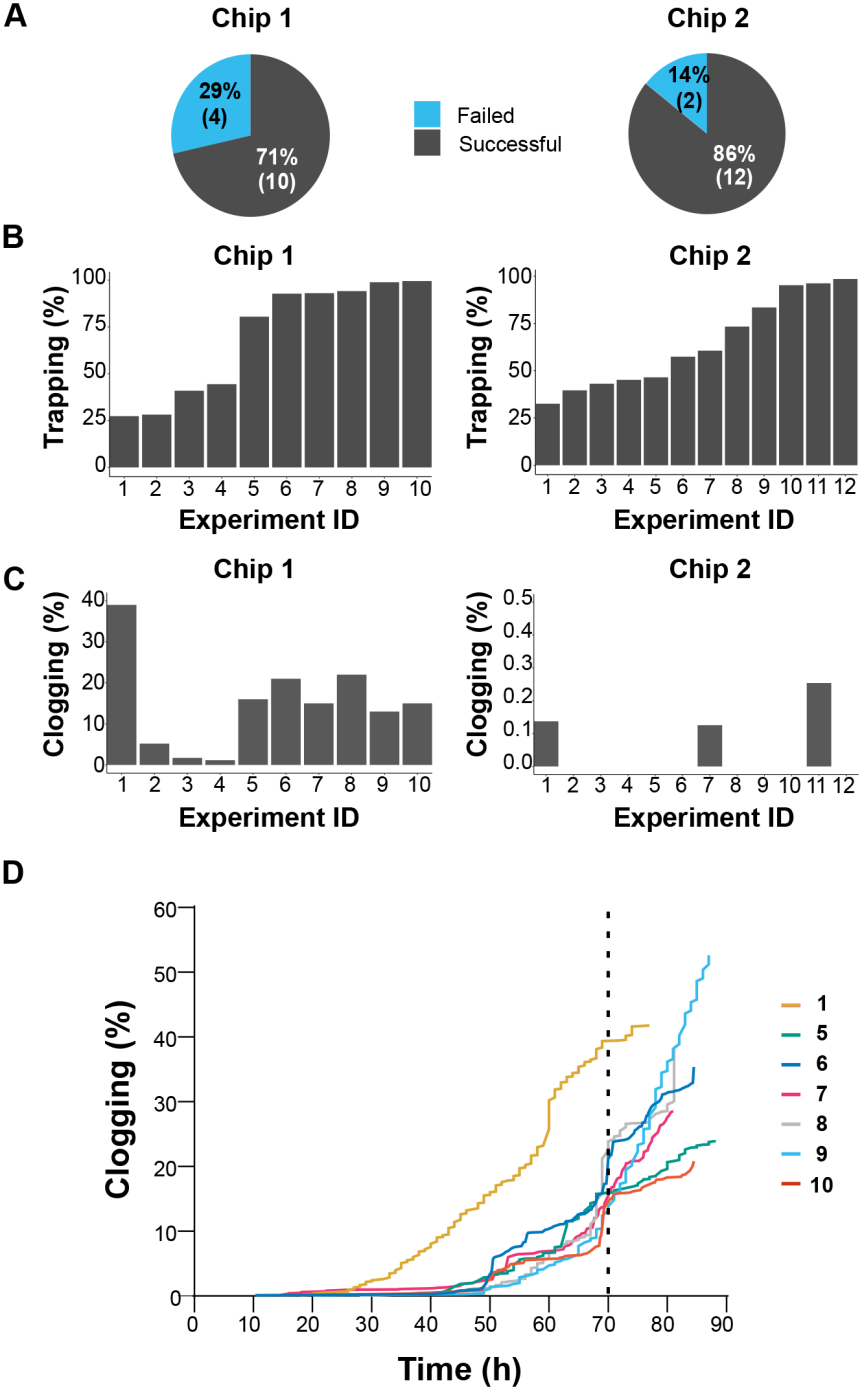
**Enhancing daughter cell removal by optimizing trap spacing.** (A) Proportion of interrupted experiments because of chip clogging and flow halting before reaching 70 h. (B,C) A total of 22 experiments with a minimum duration of 70 h were conducted using chip 1 (10 experiments), and chip 2 (12 experiments). (B) Trap occupancy rate after 20 h of the experiment. (C) Percentage of clogged traps at 70 h. Note the Y axes’ different scaling. (D) Time course of traps clogging using chip 1. Numbers indicate individual experiments, as shown in B and C (left panels). Vertical dashed line indicates the minimum experimental time required to monitor the lifespan breath of a WT *S. cerevisiae* strain population.

Having established comparable efficiency in both cell retention and downstream budding orientation, we examined the impact of trap arrangement on clogging propensity in chips 1 and 2. Microchannel clogging was assessed by monitoring the number of aborted experiments due to flow cessation before the completion of the established minimum of 70 h assay. A total of 14 comparative experiments were conducted. As depicted in Fig. 3A, clogging resulted in the termination of four out of 14 experiments using chip 1. Conversely, the likelihood of experiment failure due to clogging was reduced by half using chip 2.

While interruption of microfluidic experiments by clogging events can lead to significant time and resource waste, chip 1 still offers the potential to analyze more cells per experiment than chip 2. To settle this point, we assessed the informativeness of unclogged experiments in both chips (Fig. 3B,C). Clogging emerged as a frequent issue in chip 1, with over 70% of successful experiments exhibiting more than 15% of their traps clogged, regardless of the degree of trap occupancy. Conversely, clogging was barely detected in chip 2 experiments across a wide range of trap occupancy. Because clogging can significantly impact microfluidic RLS determinations, particularly with long-lived strains, we examined the cumulative number of obstructed traps over time. Our results show a rapid accumulation of cell clumps in chip 1, reaching up to 50% of traps clogged within 90 h (Fig. 3D).

Our data demonstrate comparable cell retention and budding orientation for both trap designs. However, chip 2's increased trap spacing effectively mitigates clogging, leading to more reliable and informative experiments. This suggests that chip 2's design is superior for long-term microfluidic RLS studies demanding uninterrupted flow.

### Reliable RLS determination

**Fig. 4.**
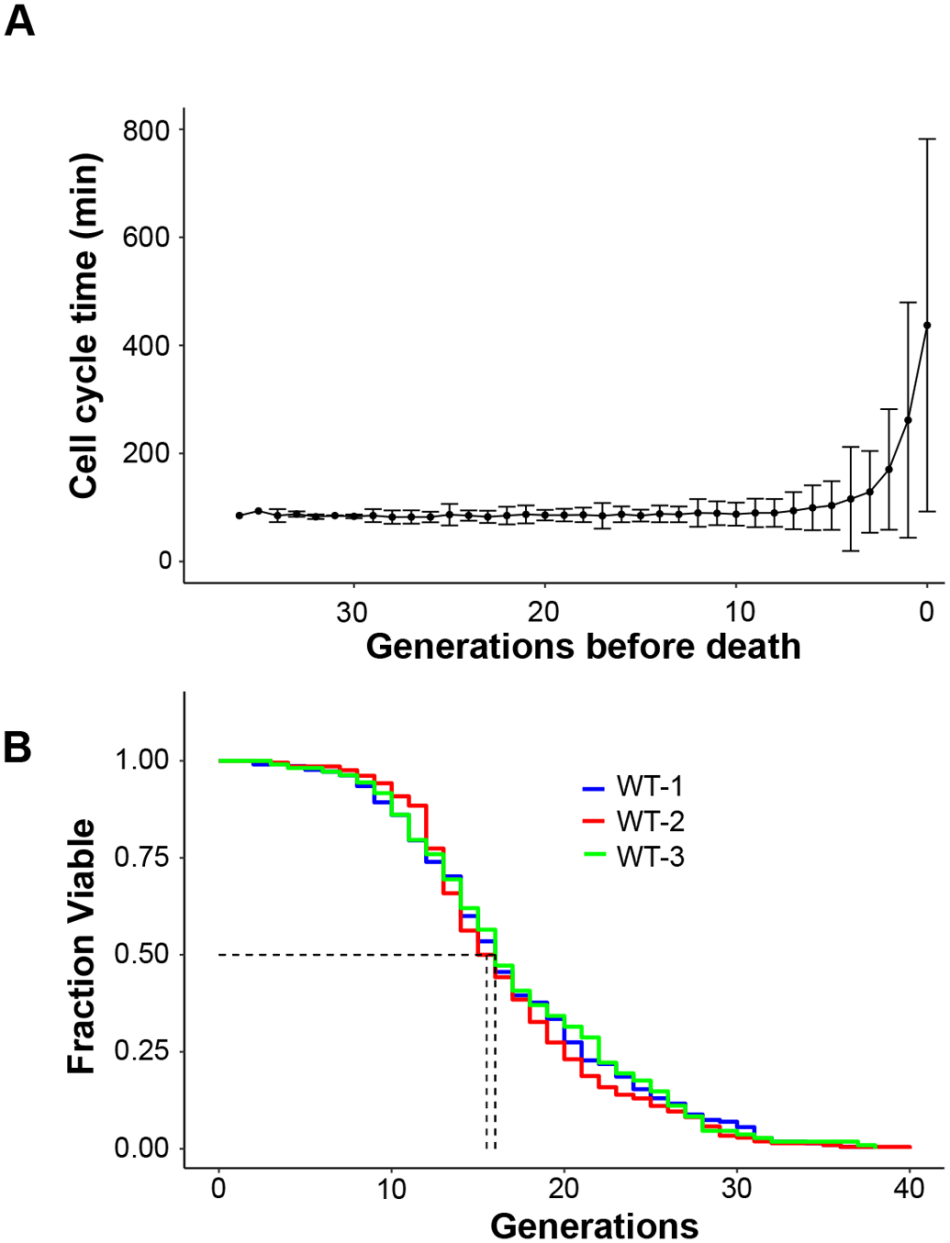
**The microfluidic device enables reliable RLS determination of a WT *S. cerevisiae* strain.** (A) The cell cycle time during the complete lifespan of 215 trapped yeast cells was tracked and manually analyzed. Cell cycle time distributions were calculated for each generation. All samples were aligned with the time of death as the reference point. Data represent the average and SD. (B) Population survival as a function of the number of generations of yeast cells for three independent experiments (WT-1, *n*=215 cells, WT-2, *n*=208 cells; WT-3, *n*=108 cells). G50 values are shown (dot lines). The obtained RLS results are not significantly different, as a Wilcoxon–Rank sum test indicates. The *P*-values for WT1-WT2, WT1-WT3 and WT2-WT3 combinations are 0.69, 0.74 and 0.52, respectively. RLS statistics and lifespan distributions are shown in Table S1 and Fig. S4, respectively.

Given chip 2's (referred to as chip from now on) superior performance, we employed it to measure the lifespan of a WT yeast strain by counting the total number of daughter cells produced by a population of trapped mother cells. The time interval between successive budding events was tracked for each cell throughout its lifespan and plotted as a function of the number of buds produced before death (Fig. 4A). As expected (Jo et al., 2015; Wang et al., 2022), the average cell cycle time exhibited minimal variation around 90 min before showing a marked increase during the final budding events. The lifespan of the WT strain was determined by plotting the fraction of viable cells against the daughter cell generation number. This analysis revealed an average G50 (the generation at which 50% of the initial cell population remains viable) of 15.8±0.3 generations across replicates (Fig. 4B; Table S1), demonstrating high reproducibility in the measurements.

The experimental RLS curve also fits well with the Weibull survival function, which is widely used to model mortality dynamics (Fig. S5) (Liu and Acar, 2018). These results highlight the robustness of the employed methodology, thereby demonstrating that the proposed design serves as a reliable tool for quantifying and comparing the lifespans of different strains.

### Characterization of the age distribution of the initially loaded cells

**Fig. 5.**
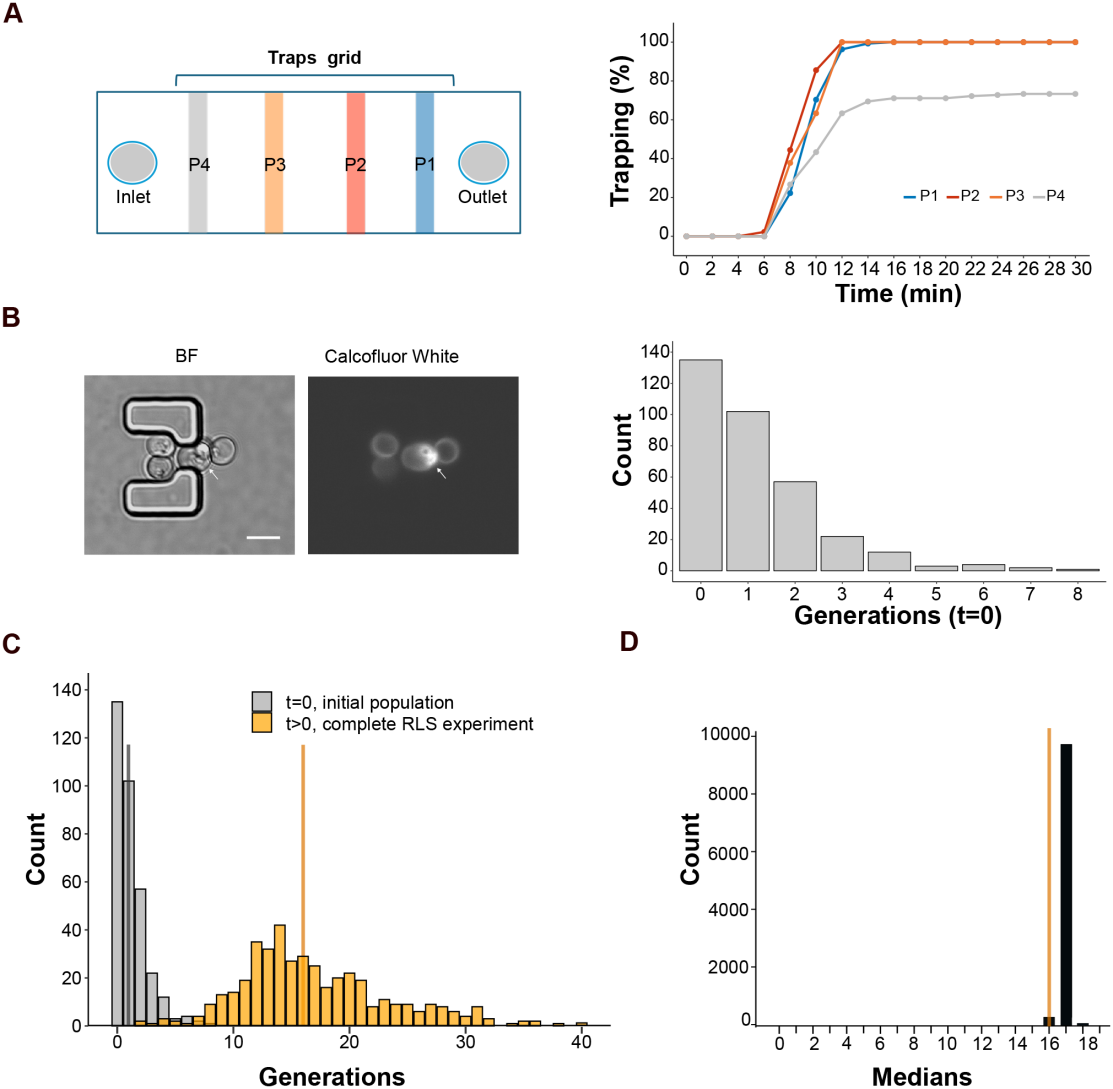
**Chip loading optimization and determination of the age distribution of the loaded population.** (A) Left: scheme of chip zones (*P*1-4) that were monitored by video microscopy during chip loading. Right: cumulative % of occupied traps during cell loading. At least 110 traps were imaged per zone. (B) Left: representative 3D projection images of yeast cells trapped in the chip and stained with calcofluor white, a white arrow points to a trapped mother cell (scale bar: 5 µm). Right: distribution of the bud scars (generations) in the yeast population at the beginning of the RLS experiment (t=0). Results of two experiments are shown (*n*=338 cells). The median value is 1. (C) Overlay of age distributions of the loaded cell population (gray, t=0, *n*=338 cells), and of the completed RLS experiment population (orange, t>0, *n*=423, WT-1 plus WT-2 experiments data), vertical lines indicate distribution's medians. (D) Distribution of median lifespans obtained when accounting for the unobserved daughters by random assignment between distributions shown in 5C. The median lifespan determined by the RLS experiment at t>0 is depicted in orange.

To standardize chip loading, getting high trap occupancy rates (70-100%) before starting RLS experiments, we employed video microscopy to monitor the cumulative number of occupied traps over time at different positions of the traps grid (Fig. 5A). We found that the maximum trapping level was reached after 14 min in downstream regions of the chip, closer to the outlet (see P1-P3 in Fig. 5A). However, traps located further upstream in the microfluidic device demonstrated a lower capture rate of approximately 70%. Therefore, to ensure optimal trapping efficiency, chips are loaded for 14 min, and monitoring positions are selected avoiding regions close to the inlet (see protocol in Materials and Methods).

We load the microfluidic chips with cells coming from exponentially growing cultures, which exhibit a heterogeneous age distribution (Lord and Wheals, 1980). Since the L-shaped traps capture cells of any age, we expect the monitored population won't be limited by virgin cells but will reflect the age distribution of an exponentially growing culture. This inclusion of older cells could lead to an underestimation of the calculated lifespan.

We therefore determined the age distribution in the populations of both, exponentially growing cells and mother cells that are trapped at the beginning of the RLS assay (t=0 s). For this, bud scars (remnants of previous budding events) (Mortimer and Johnston, 1959) were stained with calcofluor white and imaged using fluorescence microscopy. As expected (Lord and Wheals, 1980), 70% of the population of cultures used to load chips is composed of virgin daughters and first-generation mothers (Fig. S6). Z-stack analysis of loaded chips revealed a higher median value of the bud scar distribution in the initially trapped cell population (*P*-value=5.25 10^−8^, Wilcoxon–Rank sum test) (Fig. 5B). The observed difference is likely attributable to the 100 min interval during which cells are trapped within the chip but not yet subjected to imaging (see Materials and Methods). This period encompasses the previously established 90 min budding time. Therefore, to account for unobserved cell divisions that occurred before the microfluidic experiment began, we employed a bootstrapping approach to analyze the obtained data of a typical RLS experiment. We randomly selected and summed one data point from each distribution (t=0/initial and t>0/complete RLS experiment, Fig. 5C) and calculated the median value of the resulting distribution. This process was repeated 10,000 times to generate the distribution of median ages shown in Fig. 5D. Our findings suggest that the median lifespan (G50) measured by RLS experiments is approximately one generation shorter than it would be in a hypothetical scenario using only virgin cells. Therefore, a minor correction to the microfluidics RLS assay results is necessary.

### Analysis of censored cells ensures an unbiased outcome in the RLS assay

**Fig. 6.**
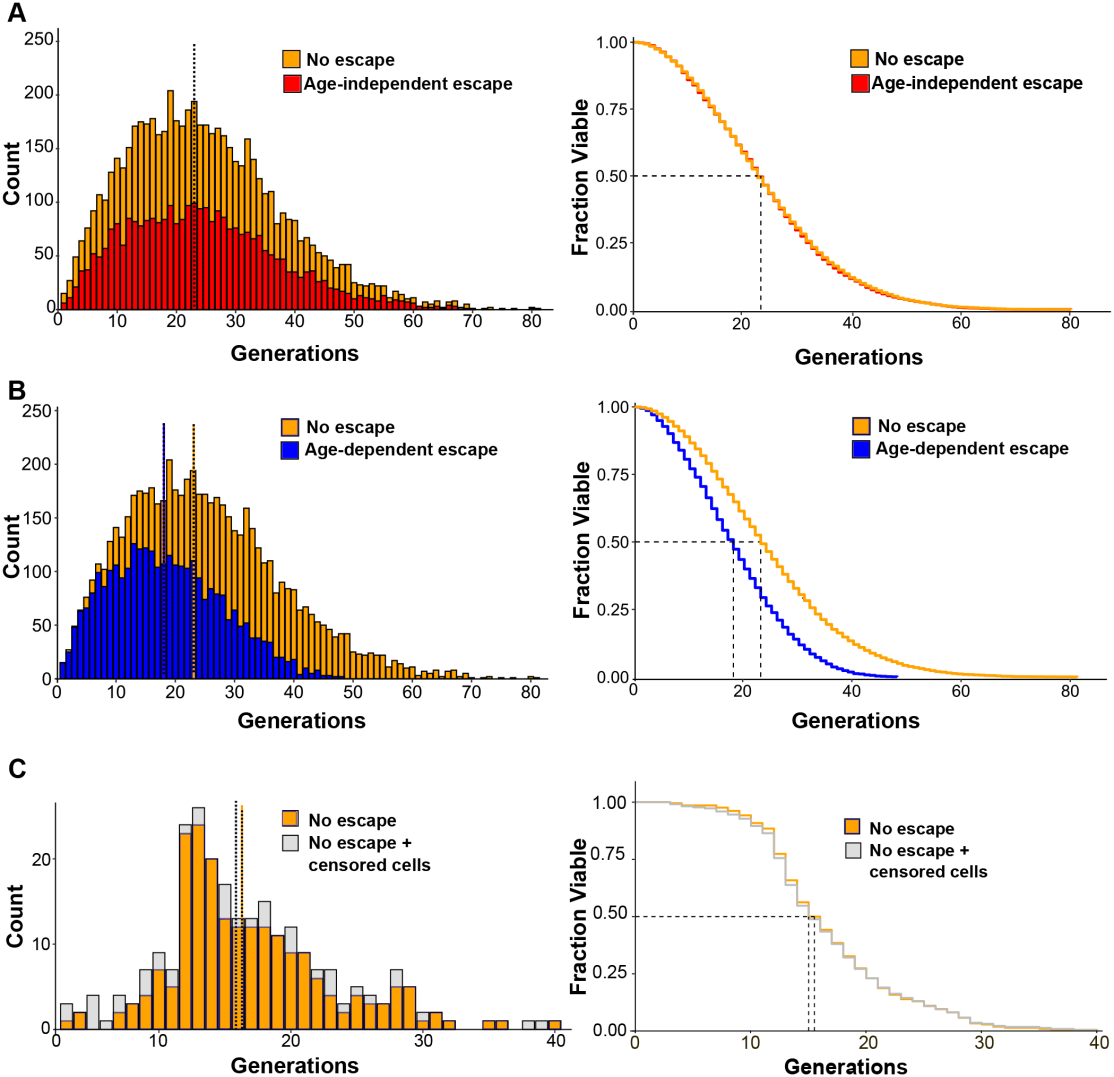
**Impact of censored cells on lifespan distributions.** (A) Left panel: comparison of lifespan distributions of two simulated cell populations: no escape from chip traps (orange) and age-independent escape (red). An initial population of 6000 cells was considered in both cases. Wilcoxon–Rank sum test *P*-value=0.7. Right panel: corresponding survival curves. Median lifespans are indicated by dotted lines. (B) Left panel: the lifespan distribution for no escaped cells (orange, same as in A) is compared to the distribution of a simulated cell population where escape probability increases proportionally with age (blue, age-dependent). Wilcoxon–Rank sum test *P*-value <2.2e^−16^. Right panel: corresponding survival curves. (C) Left panel: experimentally obtained lifespan distribution of retained cells in an RLS assay of WT cells (*n*=208 cells) compared to the distribution including both retained and censored cells (*n*=251). Wilcoxon–Rank sum test *P*-value=0.30. Right panel: corresponding survival curves.

Cell trap escape can significantly bias RLS data (Crane et al., 2014; Jo et al., 2015; Wang et al., 2022). We aimed to determine whether cell escape from our microfluidic device could lead to misinterpretations of lifespan data. Therefore, first, we simulated the lifespan distribution of a population of 6000 mother cells using the Weibull model (Liu and Acar, 2018). Three distinct scenarios were investigated: (1) no cell escape from traps, (2) random (age-independent) escape probability throughout the lifespan, and (3) age-dependent escape probability, where the likelihood of escape increases linearly with cellular aging. Our simulations revealed contrasting effects of cell escape on the lifespan distribution. When cells have a constant probability of escape throughout their lifespan (random loss), the distribution's shape and median remain remarkably similar to the ideal scenario with no escape (Fig. 6A). However, the total number of cells decreases proportionally to the escape rate.

In contrast, when escape probability increases with cell age (increasing temporal dependence), the distribution's shape is significantly altered, and the median lifespan is reduced (Fig. 6B). This finding highlights the critical importance of controlling for age-related escape in our microfluidic device for accurate lifespan measurements. To address this concern, we extended our experimental analysis by incorporating budding events from escaped (censored) cells to the retained cells distribution. By combining these data sets (Fig. 6C), we observed no significant changes in the shape or median of the lifespan distributions. In conclusion, cell loss during the RLS assay in our microfluidic device does not impact the median lifespan.

### Extended lifespan mutant RLS determination

Having verified the chip performance with a WT strain we tested its capability for monitoring long-lived strains using a *tor1* mutant. The deletion of TOR1 gene, which codes for a protein kinase part of the Target Of Rapamycin Complex 1 (TORC1), is well known to extend lifespan (Kaeberlein et al., 2005).

**Fig. 7.**
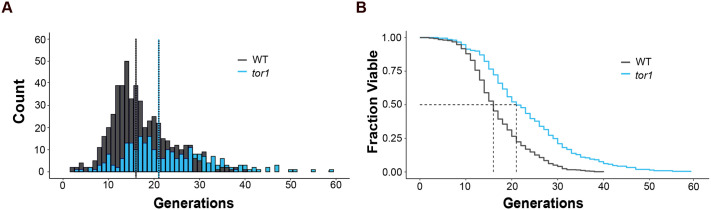
**Extended lifespan mutant RLS determination.** (A) Comparison of lifespan distributions of WT (*n*=531 cells) and *tor1* (*n*=252 cells) strains. Median lifespans are indicated by dotted lines. (B) Population survival as a function of the number of generations of WT and *tor1* cells. G50 values are indicated in the graph by dotted lines. Wilcoxon–Rank sum test *P*-value <2.2e^−16^ (RLS statistics are shown in Table S2).

After a 96 h long RLS experiment, data collection and subsequent analysis (Fig. 7, Tables S2, S3) revealed a 25% increase in the median lifespan of the *tor1* mutant compared to the WT strain, consistent with results obtained by the conventional microdissection and other microfluidics methods (Kaeberlein et al., 2005; Liu and Acar, 2018). Moreover, as expected *tor1* mutant survival curves fit the Weibull function (Kaeberlein et al., 2005; Liu and Acar, 2018) (Fig. S7). In conclusion, the chip effectively replicates the established differences in the lifespan of WT strain and the *tor1* mutant. This finding strongly validates the utility of our microfluidics platform for monitoring the lifespan of yeast.

## DISCUSSION

Microfluidics systems present a powerful tool for real-time, single-cell imaging of aging yeast cells, from birth to death. However, several technical hazards such as cell loss and chip clogging can render experiments uninformative due to lack of statistical power and heterogeneous growth conditions. We present a microfluidic device with specific features designed to address the challenges of chip cell loss and blockage. This design incorporates key strategies to ensure the passage of trapped daughter cells. First, we widened the spacing between the edge trap columns to the lateral limits of the microchannel, providing more space for daughter cells to flow through without getting trapped. Second, the design incorporates a single microchannel. This eliminates any potential pockets or intricate shapes where cells might get trapped. Third, we increased inter-traps distances by twofold compared to previous designs employing opposing L-shaped pillar traps for *S. cerevisiae* haploid cells (Jo et al., 2015). This increased spacing further facilitates the flow of daughter cells past the traps, even when large daughter cells are produced at the end of the mother cell’s lifespan. Finally, we incorporated larger buffer zones between the inlet or outlet and the traps grid. Overall, these design elements work in concert to effectively remove daughter cells while maintaining an acceptable cell retention rate and a stable and controlled-flow environment, crucial for successful RLS assays. Our results show that the clogging rate can significantly impact experiments with long-lived strains: while slower clogging allows for partial data collection, faster clogging can entirely block the chip, rendering the experiment useless (Fig. 3). This device enables the determination of reproducible lifespan curves, as demonstrated by our comparison of generations distributions using the Rank–Wilcoxon sum test (Table S1). While the observed lifespan falls within the previously reported range for haploid yeast, it resides at a relatively lower end. As detailed in Table S4, WT lifespan is sensitive to several factors. These include the specific background used, the culture medium employed, and the design of the microfluidic device itself. While efforts have been made to understand and document this inherent variability (Gao et al., 2020), it remains an actively researched area. This fact underscores the critical role of employing a reference strain in microfluidic RLS assays. The inclusion of this control facilitates robust comparisons between different strains, leading to a more accurate interpretation of lifespan data.

Furthermore, devices that trap cells without discriminating their age, like ours, may underestimate their derived lifespan due to the assumption of capturing only virgin cells. We incorporate the age distribution of the initially loaded cell population to determine the correct RLS accurately. This approach, if routinely applied, enhances the accuracy of RLS measurements.

Our microfluidic setup revealed a 22% cell escape rate (Fig. 2B). Kaplan–Meier analysis enables the integration of a censored population into a survival study as long as the mechanism of censoring (cell loss) is independent of the event of interest, cell death (Kaplan and Meier, 1958). Our experimental results demonstrate that the inclusion of censored cells does not affect the median survival of the retained cell population, indicating a stochastic pattern of cell loss throughout the RLS experiment and the plausibility of using Kaplan–Meier analysis (Fig. 6).

This work presents a microfluidic device and data analysis approach that significantly enhances the robustness and information content of yeast RLS experiments. By facilitating broader application, this approach has the potential to significantly contribute to the study of single-cell behavior and lifespan.

## MATERIALS AND METHODS

Detailed specifications of the equipment, materials and reagents are provided in Tables S5 and S6.

### Microfluidic device fabrication

#### Photolithography

Microfluidic device master molds are fabricated using photolithography technique in a cleanroom environment (class 1000, according to US Federal Standard 209D).

(1.1) Spin the negative photoresist SU-8 2005 onto a silicon wafer (4 in diameter, 525 µm thickness, Si <100> crystal plane orientation, 1-100 Ω cm^−1^ resistivity) using a spin coater machine (Speciality Coating Systems, SCS G3P-8) at 500 rpm for 10 s. Then, using an acceleration ramp of 50 rpm s^−1^ spread out the photoresist for 30 s at either 3000 rpm or 2500 rpm for 7 μm (chip1) or 8 μm (chip 2) thick master molds, respectively.
(1.2) Soft bake the wafer at 95°C for 2 min on a precision hot plate (Electronic Micro Systems, EMD-1000-1).
(1.3) Load the mask aligner with the photomask and the wafer substrate using vacuum contact. Set the exposure of UV light in 105 mJ cm^−2^.
(1.4) Remove the wafer from the mask aligner and perform a 3 min post-exposure bake at 95°C.
(1.5) Develop the photoresist for 1 min and 30 s in a bath of 100% ethyl lactate (purity ≥98%). During this time, gently shake.
(1.6) Then, rinse with 100% 2-propanol (purity ≥99.5%) and finally with distilled water. Blow dry the wafer with nitrogen (N_2_).
(1.7) Store the silicon master mold (from now on the mold) in a closed container, in the dark, at 25°C, 45% RH.
(1.8) To characterize the tallness of traps and posts, a height profile is made on each master mold with a mechanical profilometer (KLA-TENCOR, AlphaStep KLA D120).

#### Soft lithography

(2.1) Use the Sylgard 184 Silicone Elastomer Kit (Dow, USA) in a 10:1 weight ratio of elastomeric base and curing agent, respectively. Use a disposable recipient to prepare the mixture (30 g base: 3 g curing agent) and gently stir (using a disposable plastic pipette) to ensure homogeneous distribution (Fig. S8A,1). The resulting composite is PDMS (polydimethilsiloxane).
(2.2) Carefully seal the mold borders with adhesive tape to prevent leakage during PDMS pouring.
(2.3) Pour the PDMS into the mold, ensuring complete coverage and reaching a minimum of 5 mm in height, enough to facilitate chip handling. Place the mold with PDMS in a vacuum desiccator for 40 min to remove air bubbles (Fig. S8A,2-4). A glass dish or tray can be used to facilitate mold manipulation.
(2.4) Place the mold with PDMS in an oven (Bioelec, RG 41•1) and heat at 80°C for 1 h to ensure the PDMS curing process (Fig. S8A,5). Once cured, remove the adhesive tape from the mold. At this step, the mold with PDMS can be stored at room temperature.
(2.5) Carefully peel-off the PDMS stamp from the mold and keep the microchannel pattern facing up (Fig. S8A,6).
(2.6) Using a clean scalpel, cut individual chips measuring (25×10 mm, Fig. S8B,1-2). The mold is a delicate structure and susceptible to breakage. To ensure its employability, handle the mold and PDMS replica with utmost care. It is crucial to maintain the orientation of the PDMS with the trap's grid facing upwards. Moreover, avoid touching the surface of both the PDMS and the mold.
(2.7) Punch the inlet and outlet holes in the PDMS using 1 mm inner diameter biopsy punches. Position the PDMS on a stable surface and vertically align the puncher in the designated inlet area (Fig. S8B,3). Apply firm, downward pressure to achieve complete perforation of the PDMS. Verify perforation by removing the PDMS column from the punch (Fig. S8B,4-5). Carefully place the PDMS back on the work surface and remove the punch. Clean the punch with compressed air and repeat the process to create the outlet hole.
(2.8) Clean the PDMS and cover glass (#1; 24 mm×60 mm×0.13-0.16 mm) with 100% 2-propanol and dry with compressed air. Subsequently, using tweezers introduce the PDMS replicas (pattern side facing up) and cover glasses in the Harrick Plasma PDC32G-M-03 system (Fig. S8C,1-2). Apply oxygen plasma treatment for 25 s to activate the surfaces (Cabeen & Losick, 2018) (Fig. S8B,1-2). Carefully transfer the materials to a petri dish, keeping the activated surfaces facing up. Dispense a 5 μl aliquot of sterile distilled water onto the center of the PDMS (Fig. S8C,3). Then, gently place the PDMS onto the cover glass, ensuring contact between the activated surfaces. Apply gentle manual pressure to the PDMS (Fig. S8B,4) to promote firm adhesion and eliminate air gaps.
  Following successful surface activation, the water droplet deposited onto the PDMS surface will exhibit minimal spreading.
  Excessive manual pressure applied during the bonding process can induce the collapse of microfluidic posts or traps within the microfluidic chip. To mitigate these risks, it is crucial to apply gentle and controlled pressure during the bonding process. Apply pressure progressively, starting at the periphery of the bonding surfaces and gradually working towards the central region. This helps to ensure an even distribution of pressure and minimizes the potential risk of structural collapse.
(2.9) Finally, heat the assembled microfluidic chips at 95°C for 15 min (Fig. S8B,5).
(2.10) Store assembled chips into closed containers until their use.

### Yeast cell culture preparation

Yeast strains with a BY4742 background (*MATα his3*Δ*1 leu2*Δ*0 lys2*Δ*0 ura3*Δ*0*), WT and single-gene deletion strain *tor1* (Table S7) were used in this work.

A *TOR1* deletion strain of the BY4741 background (*MATa his3Δ1 leu2Δ0 mat15Δ0 ura3Δ0 tor1::kanMX4*) from the Yeast Knockout collection (Giaever et al., 2002) was mated with the WT BY4742 strain. The resulting diploid was then sporulated to obtain an haploid strain with the *TOR1* gene deleted, now in the BY4742 background. Deletion identity was verified by PCR using primers listed in Table S8.

Yeast cells are inoculated from –80°C storage on a solid YPD-agar plate for 48 h at 28°C (Table S9).

Next, individual yeast strains are grown overnight in 5 ml of fresh SC medium (Table S10) at 28°C with continuous shaking at 180 rpm. Then, the cultures are diluted to achieve an OD_600nm_ of 0.1-0.2 after at least 15 h of culturing.

The SC medium is incubated at 28°C for 2 days as a sterilization check before use. The absence of detectable contamination after this time frame indicates a high likelihood of medium sterility.

### Microfluidics live microscopy setup

Operating the microfluidic device requires a specific sequence of steps performed in a BSL-1 laminar flow cabinet (Thermo Scientific, Heraguard ECO) to prioritize sterility. Before starting, sterilization of the material as detailed in Table S11, is crucial to minimize potential biological contamination during the experiment.

(1) To prepare customized aluminum-covered tips, section disposable needles (0.8 mm diameter; 21G) to a uniform length of 1 cm. Sand their extreme distal and proximal ends to minimize surface irregularities. Finally, adhere a single square of aluminum foil with dimensions of 5×5 mm to the center region of each sectioned needle (Fig. S9A).
  Note: Aluminum foil is employed to enhance the grip of tips within the inlet and outlet orifices of the chip.
(2) Connect sequentially: (1) a 10 ml syringe, (2) a T-valve, (3) approximately 20 cm of Tygon^®^ tubing (0.8 mm internal diameter), (4) a custom-made tip.
(3) Load the perfusion pump syringes with 1% Bovine Serum Albumin (BSA) solution and set the flow rate to 1.5 mlh^−1^.
  Note: The pump program is reconfigured according to the syringe volume or flow rate.
(4) A few droplets of BSA solution are added to moisten the outlet of each microfluidic chip. Then, a second tip connected to a 20 cm length of tubing is inserted into the outlet to serve as a waste collection line (Fig. S9B,1).
(5) A similar moist procedure is undertaken at the input, with the introduction of the same tip into the port (Fig. S9B,2-3). This liquid will trap any air bubbles introduced during the replacement process, preventing them from entering the microchannel.
  Note: continuous liquid stream at the outlet confirms the successful operation (Fig. S9B,4).
(6) Sonicate the yeast cell cultures for 20 s at 10 mA to eliminate cell aggregates. Measure the culture's OD_600nm_, dilute to OD_600nm_= 0.1 and transfer them to new 10 ml syringes.
(7) To load cells, close the T-valve and connect the syringes containing the sonicated yeast to an alternate port on the T-valve. Install the new syringes within the pump and eliminate any entrapped air bubbles by purging the system towards the syringe containing the BSA solution (Fig. S9C,1-2).
(8) Reopen the T-valve to initiate the flow of the cell suspension at a constant rate of 1 mlh^−1^ for 14 min to load the chip (Fig. S9C,3).
  Note: Chip loading (OD_600nm_, flow and time) should be first optimized (see below).
  We recommend a flow rate of 1 mlh^−1^. Higher flow rates can lead to lower cell trapping.
(9) Load the syringe pump with a new assembly configuration consisting of the following components: (1) a 60 mL syringe pre-filled with RLS medium (SC medium supplemented with 0.1% BSA, final concentration); (2) a T-valve for flow direction control; (3) a one-way valve to prevent backflow; (4) a 60 cm length of tubing for fluid connection and finally (5) a customized tip (Fig. S9D).
(10) Set the pump program to a flow rate of 0.5 mlh^−1^ and connect the tip to the input, as previously described.
  Syringe and tip replacement during an experiment pose a risk of flow disruption, which can compromise experimental integrity. To avoid this, briefly increase the flow rate to 2 mlh^−1^ for a maximum of 10 min. This temporary increase can dislodge eventual clogs or air bubbles hindering the flow path.
(11) Once the proper flow is confirmed, mount the chips onto the microscope using a customized 3D-printed holder that can accommodate two chips simultaneously (Fig. S9B,4).
(12) Select optimal positions in the microscope. See “Optimization of cell loading in the microfluidic chip” section and start the recording process.
  Note: Steps 9-12 encompass a 100 min interval where cells are trapped within the chip but not yet imaged.
(13) Alternate constant flow stages and wash cycles. Maintain a constant flow rate of 0.5 mlh^−1^ and every 6.5 h include five wash cycles, each comprising two stages: (1) Flow rate of 2.5 mlh^−1^ for 10 min and (2) Flow rate of 0.5 mlh^−1^ for 20 min.
  Note: This procedure facilitates the removal of potentially accumulating cells.
(14) To ensure an uninterrupted flow of fresh culture medium throughout the experiment, a new syringe filled with fresh RLS medium is introduced when the current medium nears depletion. This is accomplished by closing the T valve and connecting the new syringes to an alternative port on the T valve, as done in steps 7 and 8 (Fig. S9C).
  Note: The temperature is controlled at 27-28°C throughout the assay.

We recommend preparing at least two additional microfluidic chips for each experiment. This practice ensures a backup in case of chip unexpected damage during the experimental setup.

### Imaging of cells in the microfluidic chip

An inverted microscope (Olympus, IX-81) coupled with a CCD camera is used. Time-lapse bright-field images are acquired every 8.5 min for a minimum of 70 h using a 40×0.95 NA air objective lens. At least 15 positions are selected in each chip using the Metamorph software workflow.

Note: Using the microscope's autofocus function is crucial for maintaining consistent image quality throughout the experiment.

### Extracting RLS and budding information from time-lapse data

The acquired images are meticulously analyzed using the image processing ImageJ software.

(1) After image acquisition, compile a movie comprising all images obtained for each position.
(2) Identify each trap in every movie.
(3) Each trapped cell is regarded as a mother cell. To streamline the workflow, categorize each cell trapped in the first 20 h of the experiment as an upstream budding, a downstream budding, or a censored cell.
(4) Manually record the frame in which a cell is trapped and the frame in which it escapes from the trap or dies.
  Note: Death is marked by a remarkable phenotypic alteration, with cells becoming flattened upon death.
(5) Register every new budding event occurring in a downstream budding cell since the cell is trapped until it dies. This rigorous documentation enables us to accurately quantify the number of daughters produced by each mother cell and ascertain the doubling time of each daughter.

### Optimization of cell loading

Microscopic observation of the chip cell loading stage is typically omitted from the standard RLS experiment procedure since it is performed in a laminar flow cabinet to prioritize sterility. Therefore, to optimize chip loading, we employed video microscopy to monitor cells' behavior upon entry into the chip.

(1) Perform the protocol described in the “Microfluidic live microscopy setup” section until step 8 and mount the chip onto the microscope.
(2) Load the cells in the chip at a rate of 1 mlh^−1^ for 30 min.
(3) As cells pass through the microfluidic chip, acquire time-lapse bright-field images every 2 min at positions located at different distances from the output (see Fig. 5A). Each selected position encompassed 45-55 traps.
  Note: the trap grid includes 173 rows (being row 1 the one closer to the output and 173 the one closer to the input). For a comprehensive analysis, we choose positions located at trap rows 160, 130, 100, and 70.
(4) Subsequently, manually determine the number of occupied traps using ImageJ software.

### Assessment of bud scars distribution

A meticulous replication of the loading conditions is undertaken to evaluate the bud scar distribution of cells initially loaded into the microfluidic device for the RLS experiment.

(1) Performed the protocol described in “Microfluidic live microscopy setup” until step 11.
(2) Load a calcofluor white filtered solution (0.5 mg ml^−1^) into a syringe of 10 ml.
(3) Close the T-valve and connect the syringe containing the calcofluor white to an alternate port on the T-valve. Adjust the new syringe within the pump and purge any introduced air toward the syringe containing the RLS medium.
(4) Reopen the T-valve allowing the calcofluor white to flow through for 20 min at a flow rate of 1.5 mlh^−1^ to stain the bud scars.
(5) Following the staining process, stop the flow, and acquire z-stack images in chip positions using a 60× N.A. oil immersion objective lens in both brightfield and fluorescence channels.
(6) Using ImageJ software, quantify bud scars on each cell.
(7) Once the bud scar distribution of cells initially loaded into the microfluidic device is determined, apply bootstrapping to integrate this distribution with the measured distribution from the RLS experiment of interest (take into account that for bootstrapping the number of cells of both distributions must be the same).
(8) Each data point of the bud scars distribution is randomly selected and added to one data point of the RLS distribution to generate a final distribution that includes the unobserved divisions. This process is repeated 10,000 times to generate the distribution of median ages (as shown in Fig. 5D). The resulting median of the medians is the corrected G50.

### Statistical analysis

A Wilcoxon–Rank sum test was used to compare pairs of datasets, each representing the number of generations of yeast cells under specific conditions as described in Figs 4, 6, and 7 using R (https://www.r-project.org/). For instance, in Fig. 7 we compared the number of generations between *tor1* mutant compared to the WT strain.

## Supplementary Material

10.1242/biolopen.060596_sup1 Supplementary information
