# Supplementary material for: Reliable replicative lifespan determination of yeast with a single-channel microfluidic chip
Source: Biol Open. 2024 Nov 26;13(11):bio060596. doi: 10.1242/bio.060596 (PMC11657196; doi:10.1242/bio.060596)
Supplement: Supplementary information [file biolopen-13-060596-s1.pdf]

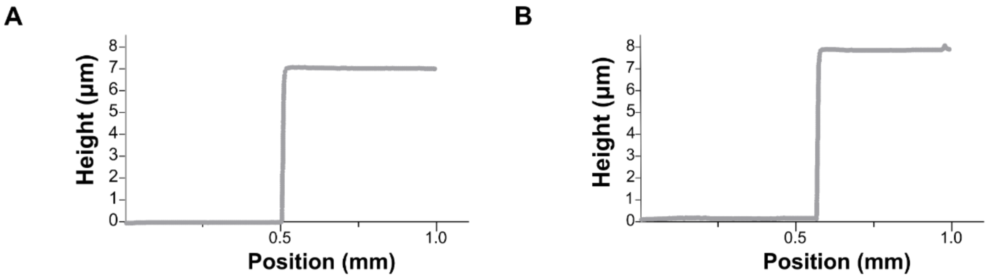

Fig. S1. Height profile. Mechanical profilometry step height measurements of SU-8 master molds of chip 1 (**A**) and chip 2 (**B**).

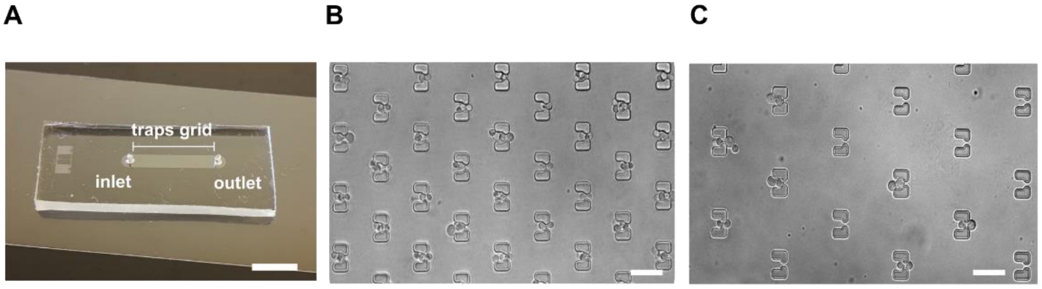

Fig. S2. Microfabrication. A. Microfluidic devices consist of a PDMS microchannel bonded to a # 1 glass coverslip. Bar, 1 cm. B and C Microscope bright field images of PDMS traps grid of chips 1 (**B**) and chip 2 (**C**). Bar, 20 µm.

Biology Open • Supplementary information

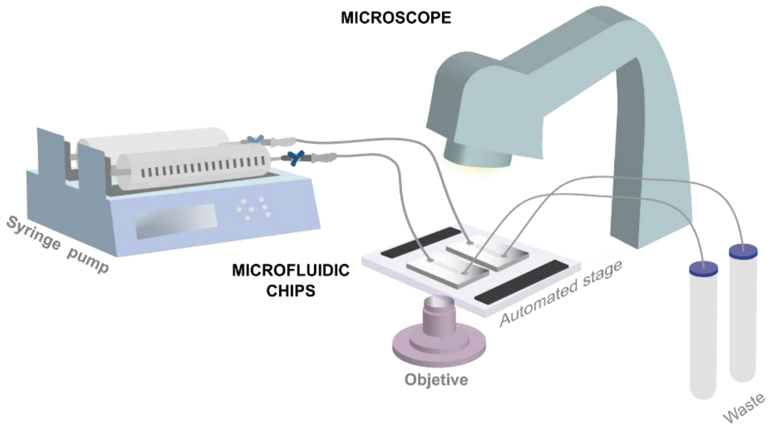

Fig S3. **Microfluidic live microscopy setup.** Schematics of the experimental setup with the microfluidic chip and a stage-automated inverted microscope.

Biology Open • Supplementary information

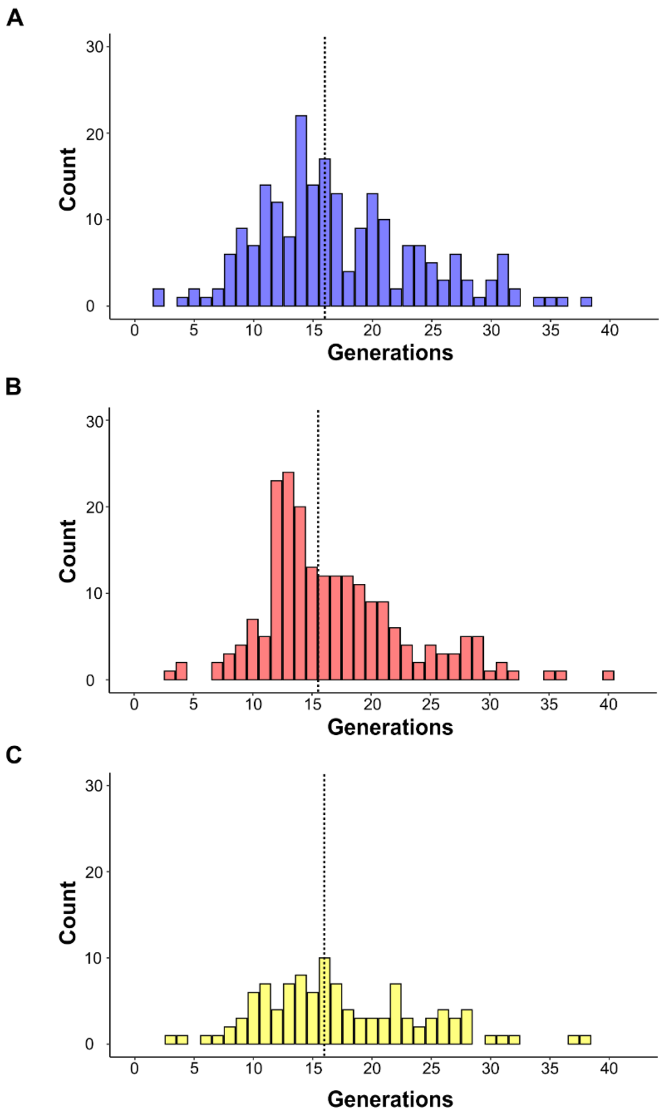

Fig. S4. Lifespan distributions of WT cells. Three independent experiments are shown. A. WT-1 B (n = 215 cells). WT-2 (n = 208 cells). C. WT-3 (n = 108 cells). Median lifespans are indicated by dotted lines. See Table S1.

Biology Open • Supplementary information

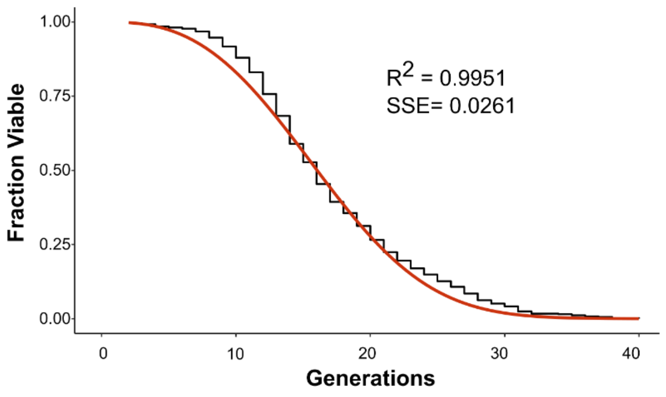

Fig. S5. Survival curves fit the Weibull function. Experimental cell survival curve (black, n = 530) was fitted to a two-parameter Weibull distribution. The red line was generated using the best-fitting parameter values extracted from the fit (SSE=sum of squared errors between black points and red curve).

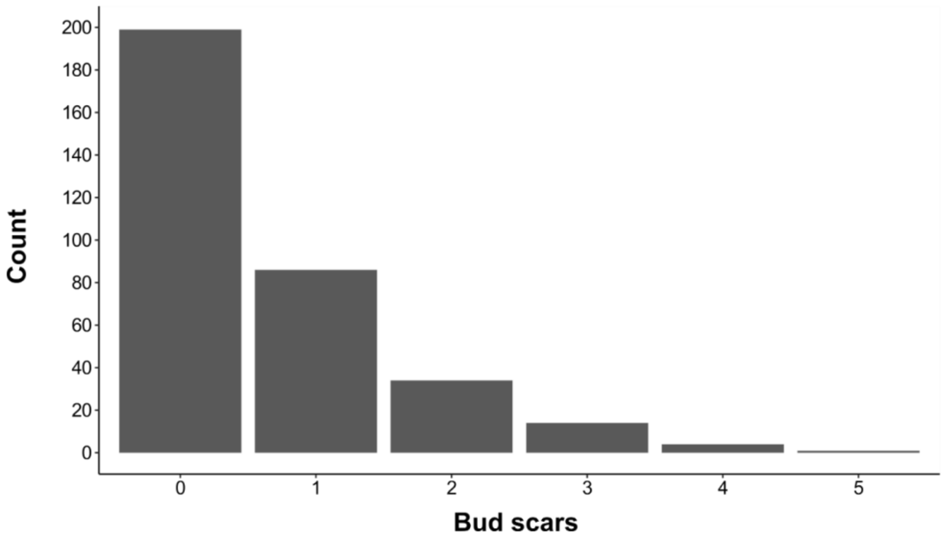

Fig. S6. Distribution of bud scars in a population of exponentially growing yeast cells. A mid-log phase yeast cell culture was incubated in a calcofluor white solution. Z-stacks images of the cells on a coverslip were taken and the number of bud scars per cell was quantified. See “Assessment of bud scars distribution” (item 7) in the Material and Methods section for details. n = 338 cells, median=0.

Biology Open • Supplementary information

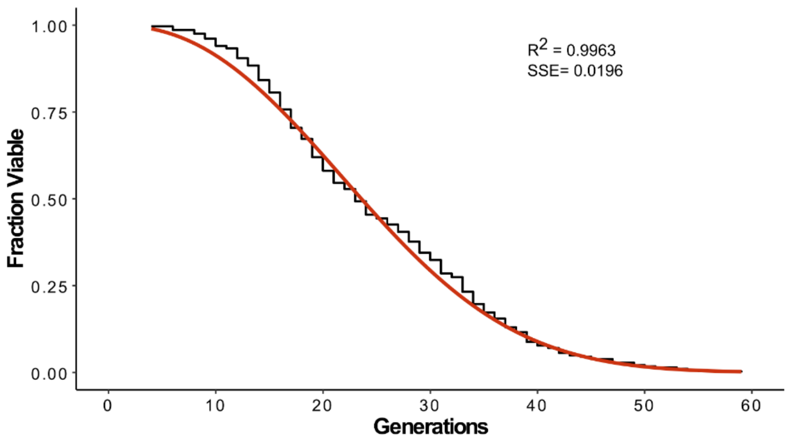

Fig. S7. *tor1* survival curve fits the Weibull function. Experimental cell survival curve of *tor1* mutant strain (black, n = 252) was fitted to two-parameter Weibull distribution (red). The red line was generated using the best-fitting parameter values extracted from the fit. n=252 cells from 2 independent experiments.

Biology Open • Supplementary information

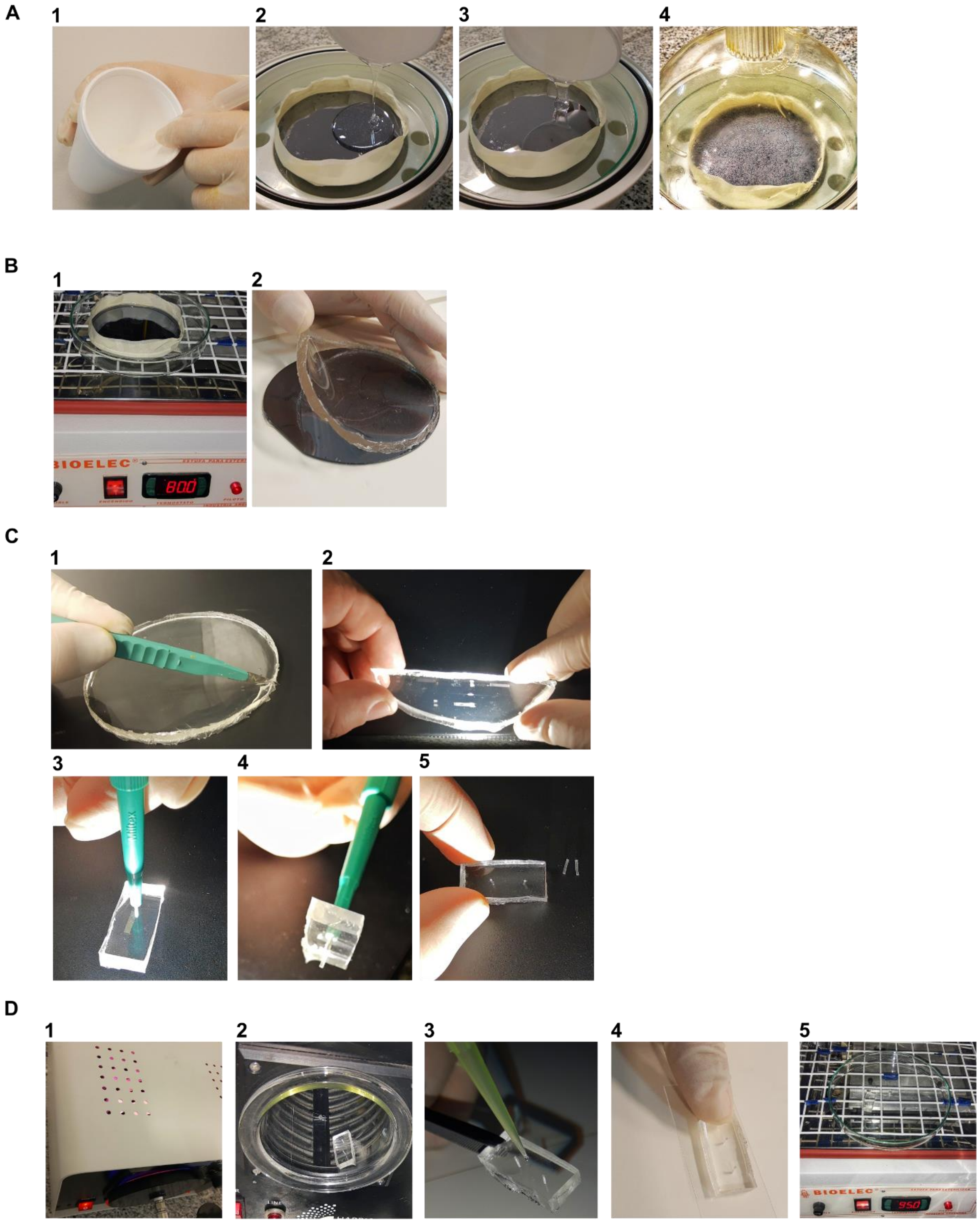

Biology Open • Supplementary information

Fig. S8. Soft lithography using PDMS. **A**. **Prepare the PDMS.** Mix the base and the curing agent thoroughly in the recommended ratio. Pour the PDMS into the mold, place it in a desiccator and applying vacuum to remove trapped air bubbles. Cure the PDMS at the recommended temperature and peel-off the PDMS replica from the mold. **B**. **Define the inlet and outlet holes** Remove PDMS around the design with a scalpel and punch holes for tubing using a biopsy puncher. **C**. **PDMS/coverslip bonding**. Activate the PDMS replica and the cover glass with O_2 plasma. Then place a drop of sterile distilled water on the PDMS slab to improve the bonding process. Assemble the device by gentle manual pressure.

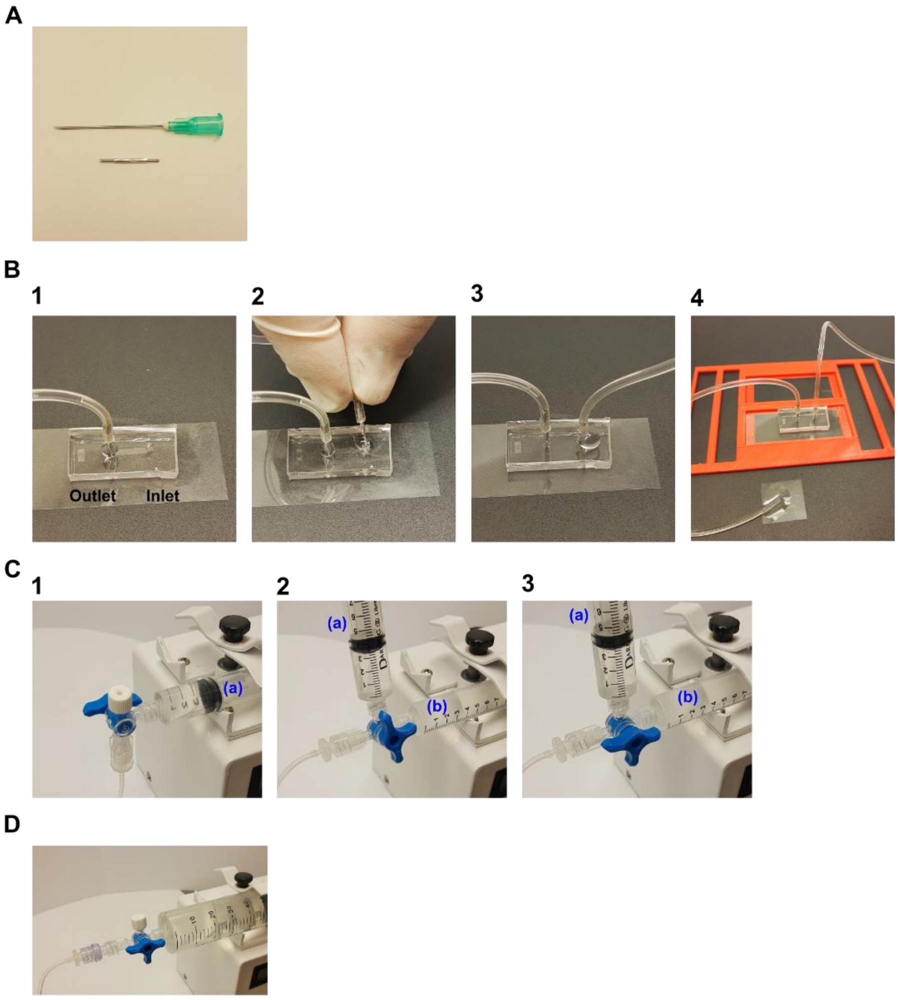

Fig. S9. Microfluidic Live Microscopy Setup. **A. Preparation of customized tips.** Use disposable needles with aluminum covers. **B. Tubing connection and flow testing**. Insert the outlet tubing. Moisten the inlet with a few drops of liquid. Insert the inlet tubing. Check for a continuous liquid stream flowing out of the outlet tubing. This confirms a successful setup. (The red colored holder in the figure is a custom 3D-printed design.). **C. Change of syringe (a) to syringe (b).** Close the T-valve, insert the new syringe (b) and place it in the pump, purge bubbles out of syringe (b) into (a). Open the T-valve to connect syringe (b) to the microfluidic chip. **D. Assembly configuration for long-term culturing** (3-4 days). Connect a 60 ml syringe, a one-way valve, a T-valve, and tubing. See details in the text.

Biology Open • Supplementary information

Table S1. Statistical parameters of three WT RLS distributions.

Experiment	Cell n°	Median	Mean	Maximum	Minimum	p value vs strain WT-1	p value vs strain WT-2	p value vs strain WT-3
WT-1	215	16	17.2	38	2		0.69	0.74
WT-2	208	15.5	16.9	40	3	0.69		0.52
WT-3	108	16	17.4	38	3	0.74	0.52	

Table S2. Statistical parameters of the *tor1* mutant and WT strain distributions.

Experiment	Cell n°	Median	Mean	Maximum	Minimum	p value vs strain *tor1*
WT	531	16	17.1	40	2	$< 2.2e^{-16}$
tor1	252	21	23.2	59	4	

Table S3. Statistical parameters of the two *tor1* mutant RLS distributions.

Experiment	Cell n°	Median	Mean	Maximum	Minimum	p value vs strain *tor1*
tor1-1	134	21.5	23.8	59	4	0.84
tor1-2	118	21	22.3	43	3	

Table S4. Reported RLS data of haploid budding yeast using microfluidic methods.

Strain	Culture media	Microfluidic device	Number of cells	RLS mean	Reference
BY4741	SC	Three bar jails	106	25.8 +/- 0,6	(Gao et al., 2020)
BY4741	SC	Opposing L shaped pillars traps	111	25.6 +/-0,7	(Gao et al., 2020)
BY4741	SC	Microchannels	121	24.9 +/-0,6	(Gao et al., 2020; Li et al., 2017)
BY4741	SC	Micropads	117	22.5 +/-0,5	(Gao et al., 2020)
BY4742	YPD	Three bar jails	100	29.3	(Liu et al., 2015)
S288C	YPD	Micropads	76	25.0	(Lee et al., 2012)
BY4741	SD	Three bar jails	-	19.2	(Durán et al., 2020)
BY4741	YPD	Micropads	40	23.6	(Zhang et al., 2012)
BY4742	YPD	Micropads	61	26	(Zhang et al., 2012)
BY4741	SC	Microcavities	99	25.3 +/- 1	(Fehrmann et al., 2013)
S288C	SC	V-shape traps	422	22.4	(Crane et al., 2014)
BY4741	YPD	Opposing L shaped pillars traps	458	24.2	(Jo et al., 2015)

Table S5. Equipment

Equipment	Source	Identifier
Spin coater machine	Speciality Coating Systems	SCS G3P-8
Precision hot plate	Electronic Micro Systems	EMD-1000-1
Mask aligner	EVG Mask Alignment Systems	EVG620
Surface profilometry system	KLA-TENCOR	AlphaStep KLA D120
Incubation oven	Bioelec	RG 41•1
Orbital shaker	Thermo Electron Corporation	SHKE435HP
Sonic dismembrator	Thermo Fisher Scientific	FB50220
Ultraviolet Crosslinker	Analytik Jena	CL-100

Biology Open • Supplementary information

Plasma cleaner	Harrick Plasma	PDC32G-M-03
Laminar flow cabinet	Thermo Scientific, BSL-1	Heraguard ECO
Microscope	Olympus	IX-81
Syringe pump	Apema	PC11UBT

Table S6. Reagents and materials.

Reagents and materials	Source	Identifier
Silicon Wafer	University Wafer	#452
Glass chrome mask	HTA Photomask	This study
Photoresist SU-8 2005	Kayaku Advanced Materials	SU-8 2005
Ethyl lactate	Sigma-Aldrich	W244007
2-propanol	Sigma-Aldrich	34863
Sylgard 184 silicone elastomer kit	Dow	2646340
Cover glass	Marienfeld	0101242
Biopsy punch with plunger	Ted Pella Miltex®	15110-10
Filters, 0,22 µm pore size	Microclar	N02025WPH
Masterflex® One-way luer check valves	Avantor	MFLX30505-92
Masterflex® Transfer Tubing, Tygon®	Avantor	MFLX06407-70
Masterflex® Adapter fittings, Female Luer to Hose Barb	Avantor	MFLX45508-00
Bovine Serum Albumin (BSA)	Sigma-Aldrich	A7906
Oxi 5 (disinfectant based on peracetic acid)	Suttley	Oxi 5
Luer lock disposable sterile syringes	Euromix	Jer60LLEUR, Jer10LLEUR
Disposable Needles	Bremen	21G
Fluorescent Brightener 28 (Calcofluor white)	Sigma Aldrich	F3543

Biology Open • Supplementary information

Table S7. Haploid strains employed in this work.

Strain	Background	Relevant genotype	Source
WT	BY4742	Wild type	EUROSCARF Y1000
tor1	BY4742	*tor1Δ* ::kanMX4	This study

Table S8. Primers used in this study.

Primer	Sequence
TOR1-F	AAT CCT AAT TTC TTG CTC AAT C
TOR1-R	AAT GGA CTG ATA TTG CCA ATA C
KanCheck-F	TGA TTT TGA TGA CGA GCG TAA T
KanCheck-Rev	CTG CAG CGA GGA GCC GTA AT

Table S9. YPD-agar**.**

Reagent	Source	Concentration (g L^{-1})	Catalog n°
Glucose	Sigma Aldrich	20	G5767
Yeast extract	Thermo Fisher	10	212750
Peptone	Thermo Fisher	20	211677
Agar	Merk	20	05040

Table S10. SC medium

Reagent	Source	Concentration (g/L)	Catalog n°
Glucose	Sigma Aldrich	20	G5767
Yeast nitrogen base	Sunrise	1.7	1500
Ammonium sulfate	Cicarelli	5	764214
Dropout mix	Sunrise	0.6	1002
Histidine	Sigma Aldrich	0.02	H6034
Tryptophan	Sigma Aldrich	0.05	T8941
Leucine	Sigma Aldrich	0.1	L8000
Uracil	Sigma Aldrich	0.02	U1128

Adenine	Sigma Aldrich	0.04	A9126
Ampicillin	Sigma Aldrich	0.1	A9518
Chloramphenicol	Sigma Aldrich	0.034	C0378
Kanamycin	Sigma Aldrich	0.05	60615

Table S11. Material sterilization.

**Material**	**Sterilization process**
BSA 1%	Filtration with nitrocellulose membrane filters (0.22 µm, pore size)
SC medium	Filtration with nitrocellulose membrane filters (0.22 µm, pore size)
Oxi 5 0.5%	Filtration with nitrocellulose membrane filters (0.22 µm, pore size)
Antibiotics	Filtration with nitrocellulose membrane filters (0.22 µm, pore size)
Tubings	4,500 Jm^{-2} UV light dosage plus perfusion of Oxi 0.5%
Adapter fittings, Female Luer to Hose Barb	Autoclave plus 4,500Jm^{-2} UV light dosage plus perfusion of Oxi 0.5%
Gloves and paper	4,500 Jm^{-2} UV light dosage
Customized aluminum-covered tips	4,500 Jm^{-2} UV light dosage

Biology Open • Supplementary information

Table S12. Data files and scripts generated in this study.
Available at https://github.com/EstradaLab-BiophotonicsGroup/RLSMicrofab/

File	Description	Figure
Trapped_cells_clasification.csv	Numbers of downstream budding, upstream budding, and censored cells in Chips 1 (n = 1,939 cells) and 2 (n = 1,978 cells)	2
Trapping_and_clogging.csv	% of occupied traps and % of clogged traps at 20h in Chips 1 and 2	3
Clogging_time_course.csv	Chip1 traps clogging time course	3
RLS_WT.csv	Number of daughter cells per mother cell of a WT strain (n = 3 experiments, n = 531 cells)	4, 5, 7, S4, S5
Cell_cycle_time.csv	Cell cycle times during the complete lifespan of 215 trapped WT cells	4
Chip_loading.csv	% of occupied traps during chip loading. At least 110 traps were imaged per zone (P).	5
Generations_bud_scars_chip.csv	Number of bud scars (generations) per cell of a WT strain mid-log phase culture loaded into the chip at the beginning of the RLS experiment (t = 0, n = 338 cells)	5
5D – Medians File.csv	Distribution of medians generated by bootstrapping using scars analysis.py	5
Scars Analysis.py	Script used for bootstrapping	5
Generations_bud_scars_flask.csv	Number of bud scars (generations) per cell of a WT of mid-log phase culture, before loading (n = 338 cells).	S6
Generations_of_censored_cells.csv	Number of daughter cells per censored mother cell (n = 43)	6
Simulated_RLS.csv	Simulated number of daughter cells per mother cell in three scenarios described in Figure 6.	6
Simulations.py	Script used to generate a distribution of daughter cells using the Weibull survival function	6
RLS_tor1.csv	Numbers of daughter cells per mother cell of a *tor1* mutant strain (n = 2 experiments, n = 252 cells)	S7, 7

Biology Open • Supplementary information

References

Crane, M. M., Clark, I. B. N., Bakker, E., Smith, S. and Swain, P. S. (2014). A microfluidic system for studying ageing and dynamic single-cell responses in budding yeast. *PLoS One* **9**, e100042.

Durán, D. C., Hernández, C. A., Suesca, E., Acevedo, R., Acosta, I. M., Forero, D. A., Rozo, F. E. and Pedraza, J. M. (2020). Slipstreaming Mother Machine: A Microfluidic Device for Single-Cell Dynamic Imaging of Yeast. *Micromachines (Basel)* **12**,.

Fehrmann, S., Paoletti, C., Goulev, Y., Ungureanu, A., Aguilaniu, H. and Charvin, G. (2013). Aging yeast cells undergo a sharp entry into senescence unrelated to the loss of mitochondrial membrane potential. *Cell Rep.* **5**, 1589–1599.

Gao, Z., Xu, J., Chen, K., Wang, S., Ouyang, Q. and Luo, C. (2020). Comparative analysis of yeast replicative lifespan in different trapping structures using an integrated microfluidic system. *Adv. Mater. Technol.* **5**, 2000655.

Jo, M. C., Liu, W., Gu, L., Dang, W. and Qin, L. (2015). High-throughput analysis of yeast replicative aging using a microfluidic system. *Proc. Natl. Acad. Sci. U. S. A.* **112**, 9364–9369.

Lee, S. S., Avalos Vizcarra, I., Huberts, D. H. E. W., Lee, L. P. and Heinemann, M. (2012). Whole lifespan microscopic observation of budding yeast aging through a microfluidic dissection platform. *Proc. Natl. Acad. Sci. U. S. A.* **109**, 4916–4920.

Li, Y., Jin, M., O'Laughlin, R., Bittihn, P., Tsimring, L. S., Pillus, L., Hasty, J. and Hao, N. (2017). Multigenerational silencing dynamics control cell aging. *Proc. Natl. Acad. Sci. U. S. A.* **114**, 11253–11258.

Liu, P., Young, T. Z. and Acar, M. (2015). Yeast Replicator: A High-Throughput Multiplexed Microfluidics Platform for Automated Measurements of Single-Cell Aging. *Cell Rep.* **13**, 634–644.

Zhang, Y., Luo, C., Zou, K., Xie, Z., Brandman, O., Ouyang, Q. and Li, H. (2012). Single cell analysis of yeast replicative aging using a new generation of microfluidic device. *PLoS One* **7**, e48275.

Biology Open • Supplementary information
